# Inflammatory Immune Responses and Gut Microbiota Changes Following *Campylobacter coli* Infection of IL-10^-/-^ Mice with Chronic Colitis

**DOI:** 10.3390/pathogens9070560

**Published:** 2020-07-11

**Authors:** Markus M. Heimesaat, Claudia Genger, Nina Biesemeier, Sigri Klove, Dennis Weschka, Soraya Mousavi, Stefan Bereswill

**Affiliations:** Institute of Microbiology, Infectious Diseases and Immunology, Charité—University Medicine Berlin, Corporate Member of Freie Universität Berlin, Humboldt-Universität zu Berlin, and Berlin Institute of Health, 12203 Berlin, Germany; claudia.genger@charite.de (C.G.); nina.biesemeier@charite.de (N.B.); sigri.klove@charite.de (S.K.); dennis.weschka@charite.de (D.W.); soraya.mousavi@charite.de (S.M.); stefan.bereswill@charite.de (S.B.)

**Keywords:** *Campylobacter coli*, murine chronic colitis, host-pathogen interaction, aged IL-10^-/-^ mice, intestinal immunopathology, bacterial colonization, gut microbiota changes, dysbiosis, enterobacterial overgrowth, intestinal and systemic immune responses

## Abstract

Human infections with the food-borne enteropathogens *Campylobacter* are progressively rising. Recent evidence revealed that pre-existing intestinal inflammation facilitates enteropathogenic infection subsequently exacerbating the underlying disease. Given that only little is known about *C. coli*–host interactions and particularly during intestinal inflammation, the aim of the present study was to survey gastrointestinal colonization properties, gut microbiota changes and pro-inflammatory sequelae upon peroral *C. coli*-infection of IL-10^-/-^ mice with chronic colitis. *C. coli* colonized the gastrointestinal tract of mice with varying efficiencies until day 28 post-infection and induced macroscopic and microscopic inflammatory changes as indicated by shorter colonic lengths, more distinct histopathological changes in the colonic mucosa and higher numbers of apoptotic colonic epithelial cells when compared to mock-infected controls. Furthermore, not only colonic innate and adaptive immune cell responses, but also enhanced systemic TNF-α secretion could be observed following *C. coli* as opposed to mock challenge. Notably, *C. coli* induced intestinal inflammatory sequelae were accompanied with gut microbiota shifts towards higher commensal enterobacterial loads in the infected gut lumen. Moreover, the pathogen translocated from the intestinal tract to extra-intestinal tissue sites in some cases, but never to systemic compartments. Hence, *C. coli* accelerates inflammatory immune responses in IL-10^-/-^ mice with chronic colitis.

## 1. Introduction

Human infections with the enteropathogens *Campylobacter* such as *C. jejuni* and *C. coli* are emerging worldwide [1]. The Gram-negative bacteria can be found in surface water and reside as commensals in the intestinal tract of many warm-blooded vertebrate species including livestock [2]. Whereas *C. jejuni* and *C. coli* share several reservoirs, the former can be isolated at high frequencies from poultry such as chicken and turkey and the latter from pig and sheep [2,3]. Following ingestion of contaminated water, milk or meat products, infected humans exhibit symptoms of varying severities after an incubation period of 2 to 5 days [4,5]. Whereas some patients complain about rather mild discomfort, others present with symptoms of acute campylobacteriosis such as fever, abdominal pain and watery or bloody and inflammatory diarrhea with mucous discharge [5,6]. *Campylobacter*-induced histopathological changes within inflamed gut tissue samples are characterized by mucosal and submucosal infiltrates consisting of innate and adaptive immune cells, crypt abscesses, focal ulcerations and erosions [7,8]. Given that *C. jejuni* and *C. coli* may induce similar disease, one cannot conclude from the clinical conditions or microscopic inflammatory sequelae to the underlying etiologic agent [2,9]. Infected individuals are usually treated symptomatically; in severe cases affecting patients with immunocompromising comorbidities including chronic inflammatory bowel disease (IBD) such as ulcerative colitis and Crohn’s disease, however, antimicrobial therapy is indicated [5,6]. The symptoms resolve without residues within two weeks in the majority of cases. In rare instances, however, post-infectious complications such as Guillain-Barré syndrome and reactive arthritis and inflammatory illnesses affecting the gastrointestinal tract, such as irritable bowel syndrome, coeliac disease, and IBD, might occur [6,10,11,12,13,14,15]. 

Recent evidence revealed that pre-existing intestinal inflammation facilitates infection with enteropathogens including *C. jejuni,* which might subsequently exacerbate the underlying disease [16,17,18,19]. Since human campylobacteriosis cases have been more frequently attributed to *C. jejuni* than to *C. coli* infections [9], research regarding the molecular mechanisms underlying *C. coli*-host mechanisms has been rather neglected during the past decades. In fact, data regarding the outcome of *C. coli* infection in pre-existing human intestinal inflammation as well as in experimental gut inflammation models are scarce. 

This prompted us to apply a murine chronic colitis model by using aged conventional interleukin (IL)-10 deficient (IL-10^-/-^) mice. With progressive aging, IL-10^-/-^ mice develop chronic colitis due to the antigenic stimuli derived from their commensal gut microbiota starting approximately by the age of 2 to 6 months depending on the housing conditions and the murine gut microbiota composition [20]. Therefore, in the present study, aged IL-10^-/-^ mice were subjected to peroral *C. coli* infection and (i) the intestinal colonization efficacies of the pathogen, (ii) the gut microbiota changes, (iii) the macroscopic and microscopic inflammatory conditions, (iv) the intestinal as well as systemic pro-inflammatory immune responses and (v) the bacterial translocation frequencies were surveyed following oral pathogen challenge.

## 2. Results

### 2.1. Gastrointestinal *Campylobacter coli* Colonization Following Peroral Infection of Aged Conventional IL-10^-/-^ mice

**Figure 1:**
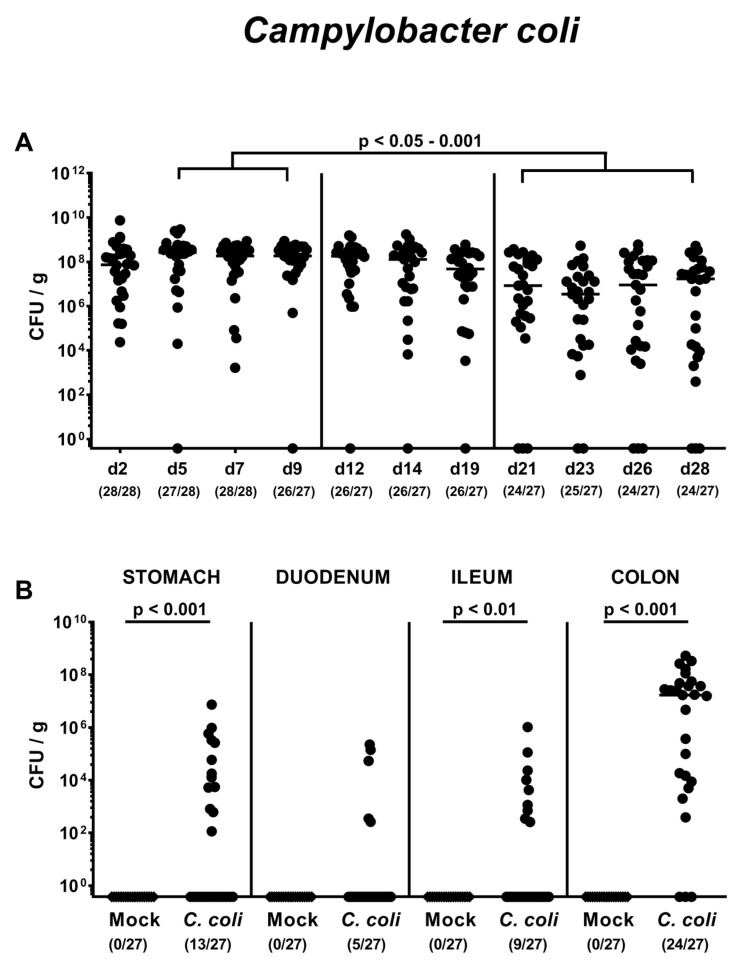
Gastrointestinal *Campylobacter coli* colonization following peroral infection of aged conventional IL-10^-/-^ mice. Ten- to 12-month-old conventional IL-10^-/-^ mice were perorally challenged with *C. coli* on day (d) 0 and d1 (circles) or received vehicle (mock controls; diamonds). (**A**) The intestinal colonization properties were surveyed over time post-infection by cultural analyses of fecal samples taken at distinct time points (expressed as colony forming units per g; CFU/g). (**B**) Upon necropsy on day 28 post-infection, *C. coli* loads were determined in distinct compartments of the gastrointestinal tract. Medians (black bars), levels of significance (*p*-values) assessed by the Kruskal–Wallis test and Dunn’s post-correction and the Mann–Whitney U test as well as numbers of culture-positive mice out of the total number of analyzed animals (in parentheses) are indicated. Data were pooled from four independent experiments.

Conventionally colonized IL-10^-/-^ mice 10 to 12 months of age were perorally infected with 10^8^ colony forming units (CFU) of a *C. coli* patient isolate on days 0 and 1 by gavage or received a mock inoculum. In order to assess intestinal colonization properties of the pathogen, we quantitated fecal *C. coli* loads over time post-infection (p.i.). As early as 24 h after the latest *C. coli* challenge, mice harbored median pathogen loads of approximately 10^8^ CFU per g feces, with individual bacterial cell counts ranging from approximately 10^4^ to 10^10^ CFU per g (Figure 1A). *C. coli* burdens assessed from day 21 until day 28 p.i. were approximately 1.5 orders of magnitude lower as compared to those determined between days 5 and 9 p.i. (*p* < 0.05–0.001; Figure 1A). At the end of the observation period on day 28 p.i., 11.1% of mice did not harbor the pathogen in their intestines anymore (Figure 1A). We further assessed *C. coli* colonization in distinct compartments of the gastrointestinal tract upon necropsy and isolated viable pathogens from stomach, duodenum, ileum and colon in 48.1%, 18.5%, 33.3% and 88.9% of cases, respectively (Figure 1B). Hence, *C. coli* was able to colonize the gastrointestinal tract of aged conventional IL-10^-/-^ mice following peroral infection but with varying efficiencies.

### 2.2. Changes in Gut Microbiota Composition Following Peroral *C. coli* Infection of Aged Conventional IL-10^-/-^ mice

**Figure 2:**
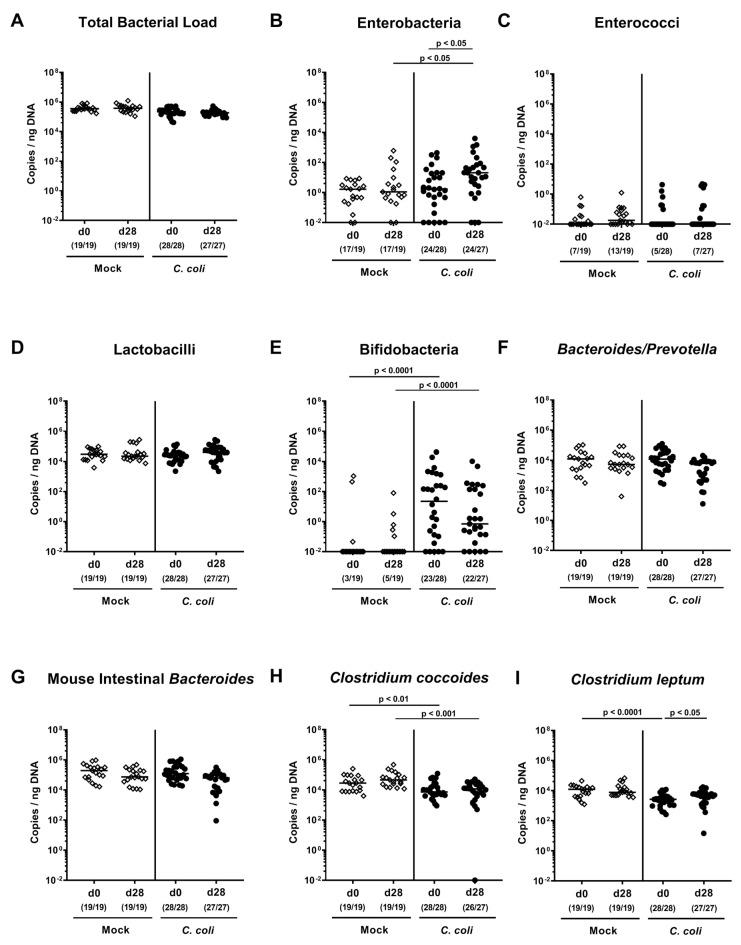
Changes in gut microbiota composition following peroral *C. coli* infection of aged conventional IL-10^-/-^ mice. Aged IL-10^-/-^ mice were perorally challenged with *C. coli* (circles) on day (d) 0 and d1 or received vehicle (mock controls; diamonds). Immediately before the first *C. coli* infection (d0) and upon necropsy (i.e., d28 post-infection), the fecal microbiota composition was surveyed by culture-independent 16S rRNA based methods quantitating the most abundant intestinal bacterial groups and genera as indicated (**A-I**) and expressed as copies per ng DNA. Medians (black bars), levels of significance (*p*-values) assessed by the Kruskal–Wallis test and Dunn’s post-correction and the numbers of 16S rRNA-positive mice out of the total number of analyzed animals (in parentheses) are indicated. Data were pooled from four independent experiments.

We further performed a comprehensive survey of potential gut microbiota changes following *C. coli* infection. Molecular 16S rRNA based analyses quantitating the most abundant intestinal bacterial groups and genera (Figure 2) revealed that 28 days following *C. coli* as opposed to mock application, approximately 2.0 and 0.5 log orders of magnitude higher gene numbers of enterobacteria and *Clostridium leptum*, respectively, could be determined in fecal samples as compared to day 0 (*p* < 0.05; Figure 2B,I). Hence, *C. coli* infection of aged conventional IL-10^-/-^ mice resulted in shifts towards higher commensal intestinal burdens of enterobacteria and *Clostridium leptum.*

### 2.3. Clinical Conditions Over Time Following Peroral *C. coli* Infection of Aged Conventional IL-10^-/-^ mice

**Figure 3:**
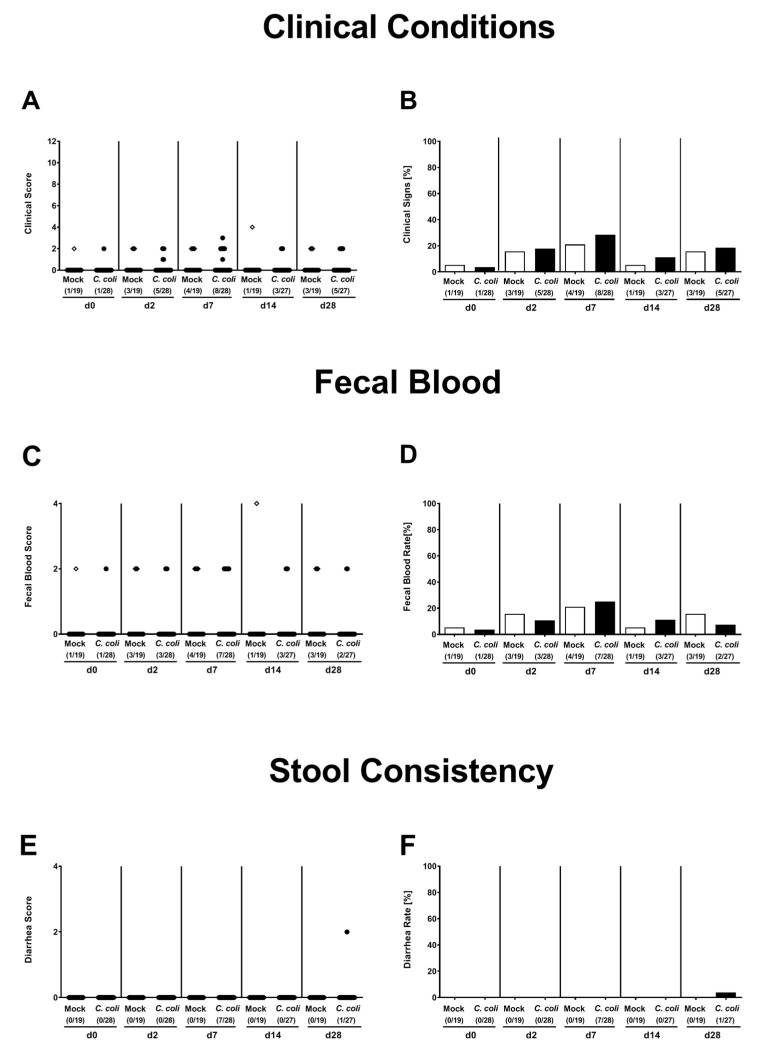
Clinical conditions over time following peroral *C. coli* infection of aged conventional IL-10^-/-^ mice. Aged IL-10^-/-^ mice were perorally challenged with *C. coli* (black symbols) on day (d) 0 and d1 or received vehicle (mock controls; white symbols). Clinical conditions of mice were surveyed over time post-infection applying a standardized clinical scoring system assessing (**A**,**B**) overall gross clinical conditions, (**C**,**D**) abundance of fecal blood and (**E**,**F**) stool consistency. Absolute clinical scores (**A**,**C**,**E**) and score-positive animals out of the total number of analyzed mice (in %; **B**,**D**,**F**) are indicated. Data were pooled from four independent experiments.

We further surveyed the clinical conditions of aged IL-10^-/-^ mice before and after *C. coli* infection applying an established clinical scoring system [21]. Before *C. coli* or mock application, only 3.6% and 5.3% of mice presented any gross signs of colitis, respectively (Figure 3A,B). When assessing clinical conditions over time p.i., only single mice displayed microscopic abundance of fecal blood, but did not suffer from diarrhea. At the end of the observation period, 18.5% of mice from the *C. coli* cohort and 15.6% of the mock counterparts exhibited rather mild gross clinical signs of colitis. Hence, *C. coli* infection did not worsen clinical conditions in aged conventional IL-10^-/-^ mice. 

### 2.4. Macroscopic and Microscopic Inflammatory Sequelae of Peroral *C. coli* Infection in Aged Conventional IL-10^-/-^ mice

**Figure 4:**
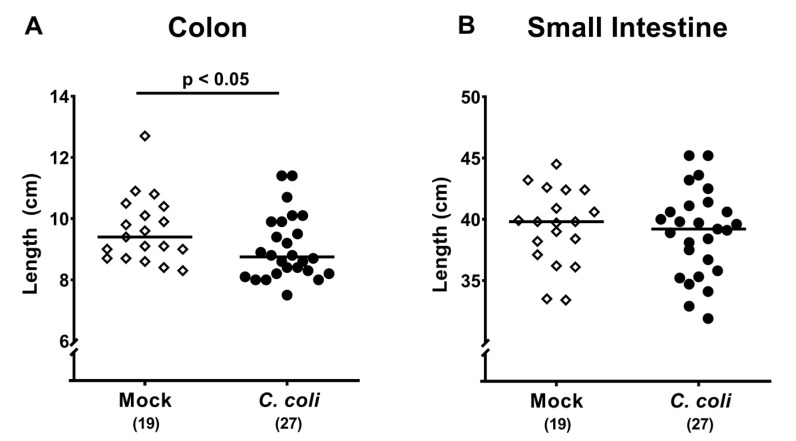
Intestinal lengths following peroral *C. coli* infection of aged conventional IL-10^-/-^ mice. Aged IL-10^-/-^ mice were perorally challenged with *C. coli* (black circles) on days 0 and 1 or received vehicle (mock controls; white diamonds). Upon necropsy (i.e., day 28 post-infection), the absolute lengths of the (**A**) colon and (**B**) small intestine were measured with the ruler (in %). Medians (black bars), levels of significance (*p*-values) assessed by the Mann–Whitney U test (**A**) and Student’s t test (**B**) and numbers of analyzed mice (in parentheses) are indicated. Data were pooled from four independent experiments.

Given that intestinal inflammation is associated with a significant shortening of the affected intestinal compartment [22,23], we measured large and small intestinal lengths upon necropsy. In *C. coli* infected mice, the absolute lengths of the colon were lower as compared to mock counterparts (*p* < 0.05; Figure 4A), whereas small intestinal lengths were comparable at day 28 p.i. (n.s.; Figure 4B). 

**Figure 5:**
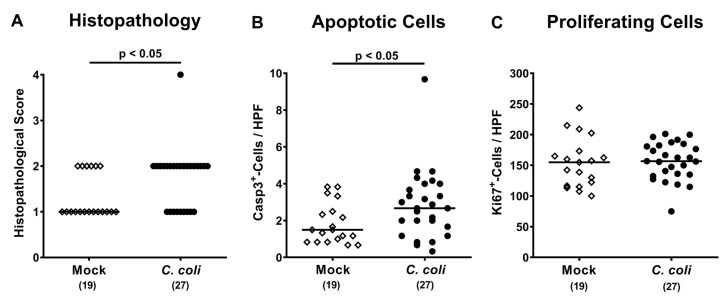
Microscopic inflammatory responses in the colon following peroral *C. coli* infection of aged conventional IL-10^-/-^ mice. Aged IL-10^-/-^ mice were perorally challenged with *C. coli* (black circles) on days 0 and 1 or received vehicle (mock controls; white diamonds). Upon necropsy (i.e., day 28 post-infection), (**A**) colonic histopathological changes were quantitatively assessed in hematoxylin and eosin (H&E) stained paraffin sections applying a histopathological scoring system (see methods). Additionally, the average numbers of colonic epithelial (**B**) apoptotic (cleaved caspase 3^+^; Casp3^+^) and (**C**) proliferating (Ki67^+^) cells were assessed microscopically from six high power fields (HPF, 400 x magnification) per animal in immunohistochemically stained large intestinal paraffin sections. Medians (black bars), levels of significance (*p*-values; assessed by the Mann–Whitney U test and Student’s t test) and numbers of analyzed mice (in parentheses) are indicated. Data were pooled from four independent experiments.

We next quantitatively assessed *C. coli* induced histopathological changes in hematoxylin and eosin (H&E) stained colonic paraffin sections by using an established histopathological scoring system [24]. Whereas mock treated mice displayed median histopathological scores of 1 indicative for rather minimal hyperplastic changes of the large intestinal mucosa [24], *C. coli* infection resulted in mild hyperplasia of the colonic mucosa and sometimes of the submucosa and mild goblet cell loss as indicated by median scores of 2 (*p* < 0.05; Figure 5A).

Since apoptosis is considered as reliable marker for the grading of intestinal inflammation [23], we further assessed the numbers of apoptotic colonic epithelial cells by in situ immunohistochemistry. On day 28 post-challenge, numbers of cleaved caspase3^+^ cells were higher in colonic epithelia of mice from the *C. coli* as compared to the mock cohort (*p* < 0.05; Figure 5B), whereas numbers of Ki67^+^ colonic epithelial cells indicative for cell proliferation and regeneration were comparable (n.s.; Figure 5C). Hence, *C. coli* infection of aged conventional IL-10^-/-^ mice with pre-existing chronic colitis resulted in both, macroscopic and microscopic inflammatory sequelae.

### 2.5. Colonic Immune Cell Responses Following Peroral *C. coli* Infection of Aged Conventional IL-10^-/-^ mice

**Figure 6:**
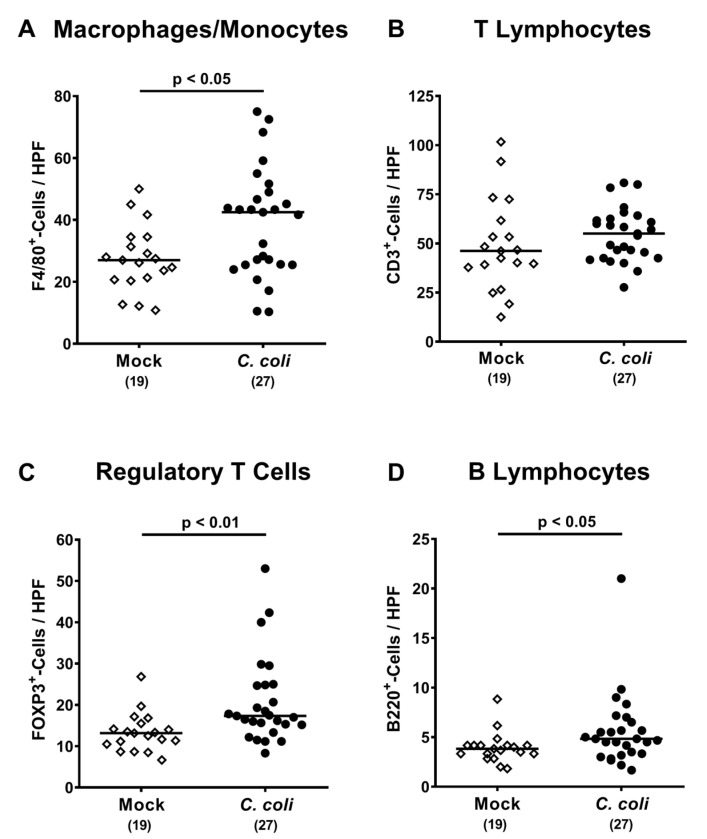
Colonic immune cell responses following peroral *C. coli* infection of aged conventional IL-10^-/-^ mice. IL-10^-/-^ mice with chronic colitis were perorally challenged with *C. coli* (black circles) on days 0 and 1 or received vehicle (mock controls; white diamonds). Upon necropsy (i.e., day 28 post-infection), the average numbers of colonic (**A**) macrophages and monocytes (F4/80^+^), (**B**) T lymphocytes (CD3^+^), (**C**) regulatory T cells (FOXP3^+^) and (**D**) B lymphocytes (B220^+^) were assessed microscopically from six high power fields (HPF, 400 x magnification) per animal in immunohistochemically stained large intestinal paraffin sections. Medians (black bars), levels of significance (*p*-values) assessed by the Student’s t test and Mann–Whitney U test and numbers of analyzed mice (in parentheses) are indicated. Data were pooled from four independent experiments.

We further investigated innate and adaptive immune cell responses upon *C. coli* infection of aged IL-10^-/-^ mice, again applying quantitative in situ immunohistochemistry. At day 28 following *C. coli* infection, higher numbers of innate immune cell populations such as F4/80^+^ macrophages and monocytes could be determined in the colonic mucosa and lamina propria as compared to mock controls (*p* < 0.05; Figure 6A), which also held true for distinct adaptive immune cell subsets such as FOXP3^+^ regulatory T cells and B220^+^ B lymphocytes (*p* < 0.01 and *p* < 0.05, respectively; Figure 6C,D), whereas CD3^+^ T lymphocyte numbers were comparable between both cohorts (n.s.; Figure 6B). Hence, *C. coli* infection of conventional IL-10^-/-^ mice with chronic colitis resulted in distinct innate and adaptive immune responses in the colon.

### 2.6. Colonic and Systemic TNF-α Secretion Following Peroral *C. coli* Infection of Aged Conventional IL-10^-/-^ Mice with Chronic Colitis

**Figure 7:**
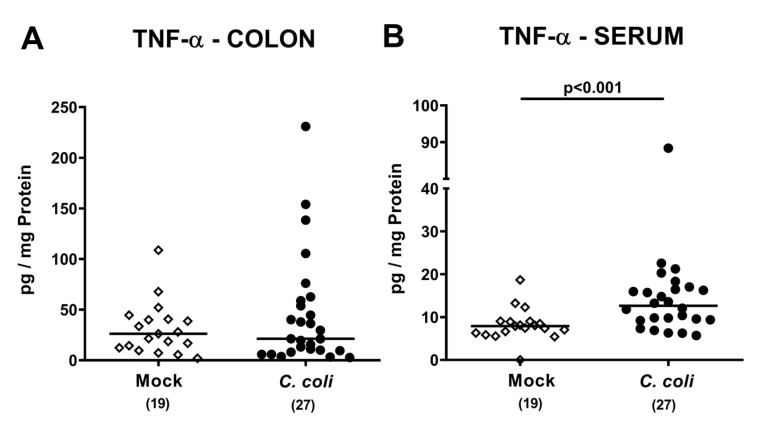
Colonic and systemic TNF-α secretion following peroral *C. coli* infection of IL-10^-/-^ mice with chronic colitis. IL-10^-/-^ mice with pre-existing chronic colitis were perorally challenged with *C. coli* (black circles) on days 0 and 1 or received vehicle (mock controls; white diamonds). Upon necropsy (i.e., day 28 post-infection), TNF-α concentrations were measured in (**A**) colonic ex vivo biopsies and in (**B**) serum samples. Medians (black bars), levels of significance (*p*-values) assessed by the Mann–Whitney U test and numbers of analyzed mice (in parentheses) are indicated. Data were pooled from four independent experiments.

We next assessed both, large intestinal and systemic tumor necrosis factor-α (TNF-α) secretion in IL-10^-/-^ mice with chronic colitis following *C. coli* infection. TNF-α concentrations were comparable in colonic ex vivo biopsies derived from *C. coli* and mock challenged mice (n.s.; Figure 7A), whereas, remarkably, TNF-α concentrations were higher in serum samples taken 28 days following *C. coli* infection as compared to mock controls (*p* < 0.001; Figure 7B). Hence, *C. coli* infection of aged conventional IL-10^-/-^ mice with chronic colitis was associated with pronounced systemic TNF-α secretion.

### 2.7. Bacterial Translocation Following Peroral *C. coli* Infection of Aged Conventional IL-10^-/-^ Mice with Chronic Colitis

**Figure 8:**
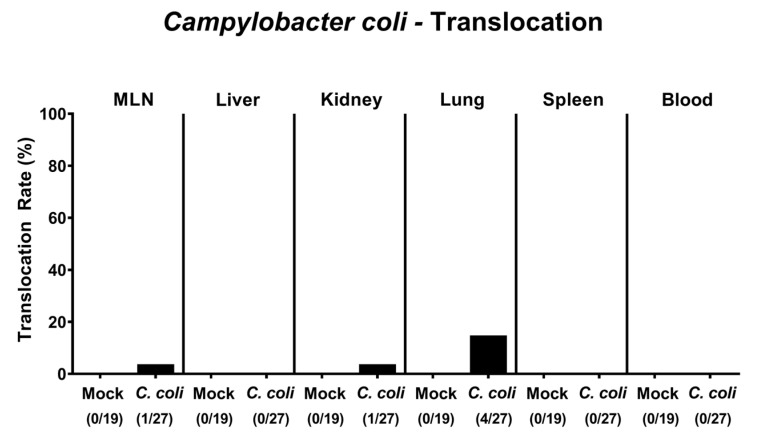
Bacterial translocation following peroral *C. coli* infection of IL-10^-/-^ mice with chronic colitis. Aged IL-10^-/-^ mice were perorally challenged with *C. coli* (black bars) on days 0 and 1 or received vehicle (mock controls; white bars). Upon necropsy (i.e., day 28 post-infection), *C. coli* were isolated from distinct compartments as indicated. The bacterial translocation rates (in %) were calculated by dividing the numbers of culture-positive samples by the total numbers of analyzed mice (in parentheses). Data were pooled from four independent experiments.

We finally addressed whether viable *C. coli* bacteria translocated from the infected intestinal tract to extra-intestinal including systemic tissue sites. In fact, *C. coli* could be isolated from ex vivo biopsies in single cases, namely, in 3.7% of mesenteric lymph nodes (MLN) and kidneys and in 14.8% of lungs taken on day 28 p.i. (Figure 8). Notably, *C. coli* could not be detected in any systemic compartments as indicated by culture-negative splenic and cardiac blood samples. Hence, *C. coli* translocated in single cases from the intestinal tract to extra-intestinal, but not to systemic compartments upon infection of IL-10^-/-^ mice with pre-existing chronic colitis.

## 3. Discussion

Previous studies revealed that patients suffering from chronic IBD are at increased risk for super-infections with enteropathogens [18,25,26,27]. Among these enteropathogens, *Campylobacter* have been frequently isolated from the intestines of super-infected patients suffering from ulcerative colitis [16,17,18,27]. Of note, *Campylobacter* super-infections worsened the outcome of the underlying chronic ulcerative colitis [19]. Despite the worldwide emerging prevalence of human campylobacteriosis our knowledge regarding the molecular mechanism of *Campylobacter*-host interactions—and of *C. coli* in particular—is very limited. This prompted us in the present study to perorally challenge aged conventional IL-10^-/-^ mice with a pre-existing chronic colitis (serving as experimental model for ulcerative colitis in humans [20]) with *C. coli* and to subsequently survey the intestinal colonization properties of the pathogen, the clinical outcome of the underlying chronic colitis until four weeks post-infection and potential *C. coli* induced shifts in the murine gut microbiota composition in addition to bacterial translocation frequencies. 

Following peroral challenge, *C. coli* colonized the intestinal tract of the aged IL-10^-/-^ mice with varying efficiencies: whereas 11.1% of mice were *C. coli*-negative at the end of the observation period, the remaining animals harbored the pathogen with median loads of approximately 10^8^ CFU per g feces but with relatively high inter-individual differences in pathogen loads ranging from 10^2^ to 10^9^ *C. coli* cells per g on day 28 p.i. In our previous study applying 3-month-old wildtype mice without intestinal inflammation, also varying *C. coli* colonization efficiencies could be observed until 3 weeks following peroral pathogen challenge [28]. In case of *C. jejuni* as opposed to *C. coli,* several bacterial factors including flagella, pili, adhesins and invasins are known to date, which contribute to successful establishment within the intestinal tract of the vertebrate host [29,30]. 

Four weeks following *C. coli* infection, IL-10^-/-^ mice with chronic colitis did neither display more frequent nor more severe clinical signs of intestinal inflammation as compared to mock-infected counterparts. However, in *C. coli* infected mice, slightly shorter colonic lengths could be observed as compared to mock control animals indicative of more severe intestinal inflammation resulting in shrinkage of the affected intestinal compartment in the former. This also held true for *C. coli* infected conventional wildtype mice as shown in our previous study [28]. Inflammatory sequelae of infection could also be observed at microscopic levels given that upon *C. coli* as compared to mock challenge moderate versus mild histopathological changes within the colonic mucosa could be detected that were accompanied by higher numbers of apoptotic colonic epithelial cells in the former versus the latter. In support, human microbiota associated IL-10^-/-^ mice without chronic colitis displayed more pronounced colonic apoptosis three weeks following peroral *C. coli* as compared to mock application [31]. In the human microbiota associated IL-10^-/-^ mice, the gut microbiota had been depleted as early as 3 weeks post-partum (i.e., upon weaning) in order to avoid colitis development due to the antigenic stimuli derived from the commensal gut microbiota [31]. These secondary abiotic IL-10^-/-^ mice were re-associated with a human microbiota by oral fecal microbiota transplantation and one week later perorally challenged with *C. coli* for unraveling the triangle relationship between the pathogen, the human gut microbiota and the vertebrate host immunity. Of note, the establishment of the human gut microbiota within the IL-10^-/-^ mice per se did not result in intestinal inflammation. Hence, all observed inflammatory responses were due to *C. coli* challenge [31].

The here observed *C. coli* induced inflammatory changes in the large intestinal tract were accompanied by enhanced innate and adaptive immune cell responses as indicated by higher numbers of macrophages and monocytes as well as of regulatory T cells and B lymphocytes in the colonic mucosa and lamina propria. Comparable immune cell responses upon *C. coli* challenge have been described in human microbiota associated IL-10^-/-^ mice without pre-existing colitis by us recently [31]. Unexpectedly, pro-inflammatory TNF-α secretion was comparable in the large intestinal tract 28 days following *C. coli* as compared to mock challenge of IL-10^-/-^ mice with chronic colitis, whereas systemically (i.e., in serum samples), higher TNF-α concentration could be assessed in the former versus the latter. We therefore addressed whether viable *C. coli* might have translocated from the large intestinal lumen through the leaky gut epithelial barrier to systemic compartments but were unable to isolate any viable pathogenic cells from spleen or blood. One needs to take into consideration, however, that soluble cell wall constituents of *C. coli* such as lipooligosaccharide (LOS) might have gained access to the circulation and have been responsible for the observed systemic TNF-α secretion. Of note, also in our previous study applying human microbiota associated IL-10^-/-^ mice without pre-existing colitis, *C. coli* induced systemic TNF-α secretion, whereas splenic and blood samples were all culture-negative for *C. coli* [31]. Nevertheless, in our actual study, *C. coli* could be cultured from extra-intestinal ex vivo biopsies such as kidneys and lungs in single cases. 

Given pathogen–commensal bacterial interactions and potential gut microbiota shifts during inflammatory conditions [32], we further performed a comprehensive survey of the gut microbiota compositions during *C. coli* infection of aged IL-10^-/-^ mice. Within 28 following *C. coli* as opposed to mock challenge, slightly higher (i.e., 0.5 log) fecal loads of *Clostridium leptum* could be observed, whereas the increase in commensal enterobacteria was even more pronounced (i.e., approximately 2.0 log). In line, acute and chronic inflammatory conditions of the murine large and small intestines have been shown to be accompanied with a marked intestinal dysbiosis characterized by increases in commensal Gram-negative bacteria including enterobacteria. The enterobacterial overgrowth of the inflamed intestinal lumen further perpetuates the underlying inflammatory morbidities due to Toll-like receptor-4 (TLR-4) dependent signaling of the bacterial lipopolysaccharides (LPS) and LOS mounting in a vicious pro-inflammatory cycle [22,33,34,35,36,37]. In line, commensal enterobacteria such as *E. coli* were shown to accumulate in the inflamed intestines of IBD patients, subsequently translocating via microlesions and ulcerations and thereby worsening the immunopathological scenario [38,39]. Hence, the observed increases in intestinal enterobacteria during *C. coli* infection may be considered as a by-standing parameter for the pathogen induced inflammation.

## 4. Materials and Methods

### 4.1. Ethics Statement

All described animal experiments were conducted according to the European Guidelines for animal welfare (2010/63/EU) after being approved by the commission for animal experiments (“Landesamt für Gesundheit und Soziales”, LaGeSo, Berlin, registration number G0247/16). The clinical conditions of mice were assessed twice daily.

### 4.2. Mice

IL-10^-/-^ mice (C57BL/6J background) were bred and raised under specific pathogen-free conditions in the identical unit of the Forschungseinrichtungen für Experimentelle Medizin (Charité–University Medicine Berlin). Under standard conditions (i.e., 22–24 °C temperature, 55 ± 15 % humidity, 12h light /12h dark cycle), mice were maintained in autoclaved cages covered by filter tops within an experimental semi-barrier (accessible only with a lab coat, overshoes, caps, face masks, and sterile gloves). Mice had free access to both, autoclaved chow (food pellets: ssniff R/M-H, V1534-300, Sniff, Soest, Germany) and tap water (ad libitum). Ten-to-12-month-old, age-and-sex-matched IL-10^-/-^ mice with a conventional gut microbiota were included into the infection studies.

### 4.3. *C. coli* Infection, Colonisation and Translocation

The used *C. coli* strain was initially isolated from a diseased patient with bloody diarrhea (kindly provided by Dr. Torsten Semmler, Robert Koch Institute Berlin, Berlin, Germany). On days 0 and 1, mice were perorally infected with 10^8^ CFU of *C. coli* by gavage in a total volume of 0.3 mL phosphate buffered saline (PBS; Thermo Fisher Scientific, Waltham, MA, USA) as described earlier [28,31], whereas the mock control cohort received vehicle (i.e., PBS) by gavage. 

Intestinal colonization properties were assessed by quantitating *C. coli* loads in fecal samples taken over time p.i. and additionally, in luminal samples derived from distinct compartments of the gastrointestinal tract such as the stomach, duodenum, ileum and colon on the day of necropsy (i.e., day 28 p.i.) as reported previously [28,31]. Briefly, serial dilutions of respective samples were plated onto Columbia agar plates with 5 % sheep blood and Karmali agar plates (both from Oxoid, Wesel, Germany) and incubated under microaerophilic conditions in a jar for 48 h at 37 °C. To survey for bacterial translocation, ex vivo biopsies were collected from the MLN, liver, kidneys, lungs and spleen, homogenized in sterile PBS and plated onto respective culture plates. Furthermore, *C. coli* were isolated from cardiac blood as described previously [31]. The *C. coli* detection limit was approximately ≈ 100 CFU per g.

### 4.4. Analyses of the Gut Microbiota Composition

For a comprehensive survey of changes in the microbiota composition p.i., fecal samples were subjected to molecular gut microbiota analyses. Therefore, the total genomic DNA was extracted from the fecal samples as described previously [34]. In brief, the DNA was quantitated by using Quant-iT PicoGreen reagent (Invitrogen, Carlsbad, CA, USA) and the concentration adjusted to 1 ng per µL. The main bacterial groups abundant in the commensal gut microbiota of mice were assessed by quantitative real-time polymerase chain reaction (qRT-PCR) with species-, genera- or group-specific 16S rRNA gene primers (Tib MolBiol, Berlin, Germany) as described previously [40,41].

### 4.5. Clinical Conditions

Immediately before and after *C. coli* application, the clinical conditions of mice were surveyed applying a standardized, cumulative clinical score (maximum 12 points), assessing the gross clinical aspect (0: normal; 1: ruffled fur; 2: less locomotion; 3: isolation; 4: severely compromised locomotion, pre-final aspect), the occurrence of blood in feces (0: no blood; 2: microscopic detection of blood by the Guajac method using Haemoccult, Beckman Coulter/PCD, Germany; 4: macroscopic blood visible), and diarrhea (0: formed feces; 2: pasty feces; 4: liquid feces), as described previously [21]. The relative rates of respective parameters were calculated by dividing the number of score-positive cases by the total number of analyzed mice.

### 4.6. Sampling Process

On the day of necropsy (i.e., day 28 p.i.), mice were sacrificed by CO_2_ asphyxiation. Ex vivo biopsies were taken under sterile conditions from liver, kidneys, spleen, lungs, MLN and colon, in addition to luminal samples from stomach, duodenum, ileum and colon. Blood was collected by heart puncture. Colonic samples were taken from each mouse in parallel for microbiological, immunohistopathological and immunological analyses. The absolute lengths of the large and small intestines were measured with a ruler.

### 4.7. Histopathological Scoring

The histopathological changes in the large intestines were quantitatively assessed in colonic ex vivo biopsies that were immediately fixed in 5 % formalin and embedded in paraffin. Therefore, a standardized histopathological scoring system was used as described elsewhere [24]. In brief, score 1: minimal inflammatory cell infiltrates in the mucosa with intact epithelium. Score 2: mild inflammatory cell infiltrates in the mucosa and submucosa with mild hyperplasia and mild goblet cell loss. Score 3: moderate inflammatory cell infiltrates in the mucosa with moderate goblet cell loss. Score 4: marked inflammatory cell infiltration into in the mucosa and submucosa with marked goblet cell loss, multiple crypt abscesses and crypt loss.

### 4.8. Immunohistochemistry

In situ immunohistochemical analyses were carried out in formalin fixed and paraffin embedded colonic ex vivo biopsies as stated earlier [36,42,43,44]. For detection of apoptotic and proliferating colonic epithelial cells, macrophages/monocytes, T lymphocytes, regulatory T cells, and B lymphocytes, 5 µm colonic paraffin sections were stained with primary antibodies directed against cleaved caspase-3 (Asp175, Cell Signaling, Beverly, MA, USA, 1:200), Ki67 (TEC3, Dako, Glostrup, Denmark, 1:100), F4/80 (# 14-4801, clone BM8, eBioscience, San Diego, CA, USA, 1:50), CD3 (#N1580, Dako, 1:10), FOXP3 (clone FJK-165, #14-5773, eBioscience, 1:100) and B220 (No. 14-0452-81, eBioscience; 1:200), respectively. Positive-stained cells were assessed by light microscopy, and the average number within at least six high power fields (HPF, 0.287 mm^2^, 400 times magnification) was recorded by an independent investigator.

### 4.9. Pro-Inflammatory Cytokine Measurements in Large Intestinal and Systemic Compartments

Colonic ex vivo biopsies were cut longitudinally, washed in PBS, and strips of approximately 1 cm^2^ colonic tissue were placed in 24-flat-bottom well culture plates (Thermo Fisher Scientific, Waltham, MA, USA) containing 500 µL serum-free RPMI 1640 medium (Thermo Fisher Scientific, Waltham, MA, USA) supplemented with penicillin (100 U/mL) and streptomycin (100 µg/mL; Biochrom, Berlin, Germany). After incubation for 18 h at 37 °C, colonic culture supernatants as well as serum samples were tested for TNF-α by the Mouse Inflammation Cytometric Bead Array (CBA; BD Biosciences, Heidelberg, Germany) on a BD FACSCanto II flow cytometer (BD Biosciences, Heidelberg, Germany).

### 4.10. Statistical Analysis

Medians and levels of significance were calculated applying the Student’s t test and Mann–Whitney U test (GraphPad Prism v8, USA) for pairwise comparisons of normally distributed and not normally distributed data, respectively. For multiple comparisons, the one-sided ANOVA with Tukey post-correction was assigned for normally distributed data and the Kruskal–Wallis test with Dunn’s post-correction for not normally distributed data. Two-sided probability (*p*) values ≤ 0.05 were considered significant. Data were pooled from four independent experiments.

## 5. Conclusions

For the first time, we here show that *C. coli* induces intestinal and systemic inflammatory responses in conventional aged IL-10^-/-^ mice with pre-existing chronic colitis. Further studies are needed to unravel the molecular mechanism underlying *C. coli*-host interactions in more detail. Given the importance of the gut luminal milieu and its metabolomic features that are determined by the gut microbiota composition, in contributing to “intestinal health” [45], we are currently assessing the immunomodulatory (i.e., anti-oxidative, anti-inflammatory) properties of distinct probiotic and prebiotic compounds as therapeutic and/or preventive strategies to combat *Campylobacter* induced inflammatory responses in the vertebrate host with intestinal inflammatory comorbidities.

## Abbreviations

CBA	Cytometric Bead Array
CFU	colony-forming units
d	day
H&E	hematoxylin and eosin
HPF	High power fields
IBD	inflammatory bowel disease
IL	interleukin
LOS	lipooligosaccharide
LPS	lipopolysaccharide
MLN	mesenteric lymph nodes
n.s.	not significant
PBS	phosphate buffered saline
*p*-value	probability value
p.i.	post-infection
qRT-PCR	quantitative real-time polymerase chain reaction
TNF	tumor necrosis factor
TLR-4	Toll-like receptor-4
